# Optimization of Extraction Conditions and Characterization of Volatile Organic Compounds of *Eugenia klotzschiana* O. Berg Fruit Pulp

**DOI:** 10.3390/molecules27030935

**Published:** 2022-01-29

**Authors:** Ana P. X. Mariano, Ana L. C. C. Ramos, Afonso H. de Oliveira Júnior, Yesenia M. García, Ana C. C. F. F. de Paula, Mauro R. Silva, Rodinei Augusti, Raquel L. B. de Araújo, Júlio O. F. Melo

**Affiliations:** 1 Departamento de Alimentos, Faculdade de Farmácia, Campus Belo Horizonte, Universidade Federal de Minas Gerais, Belo Horizonte 31270-901, Brazil; anapaula.xavier.mariano@hotmail.com (A.P.X.M.); analuizacoeli@gmail.com (A.L.C.C.R.); raquel@bromatologiaufmg.com.br (R.L.B.d.A.); 2 Departamento de Ciências Exatas e Biológicas, Campus Sete Lagoas, Universidade Federal de São João Del-Rei, Sete Lagoas 36307-352, Brazil; afonsohoj@gmail.com (A.H.d.O.J.); jenny_thesiba@hotmail.com (Y.M.G.); 3 Departamento de Ciências Agrárias, Instituto Federal de Educação, Ciência e Tecnologia de Minas Gerais, Campus Bambuí, Bambui 38900-000, Brazil; ana.paula@ifmg.edu.br; 4 Departamento de Nutrição, Pontifícia Universidade Católica de Minas Gerais, Belo Horizonte 30640-070, Brazil; mauroramalhosilva@yahoo.com.br; 5 Departamento de Química, Campus Belo Horizonte, Universidade Federal de Minas Gerais, Belo Horizonte 35702-031, Brazil; augusti.rodinei@gmail.com

**Keywords:** Myrtaceae, headspace solid-phase microextraction, Cerrado, aroma, volatile organic compounds

## Abstract

*Eugenia klotzschiana* O. Berg is a native species to the Cerrado biome with significant nutritional value. However, its volatile organic compounds (VOCs) chemical profile is not reported in the scientific literature. VOCs are low molecular weight chemical compounds capable of conferring aroma to fruit, constituting quality markers, and participating in the maintenance and preservation of fruit species. This work studied and determined the best conditions for extraction and analysis of VOCs from the pulp of *Eugenia  klotzschiana* O. Berg fruit and identified and characterized its aroma. Headspace solid-phase microextraction (HS-SPME) was employed using different fiber sorbents: DVB/CAR/PDMS, PDMS/DVB, and PA. Gas chromatography and mass spectrometry (GC-MS) were employed to separate, detect, and identify VOCs. Variables of time and temperature of extraction and sample weight distinctly influenced the extraction of volatiles for each fiber. PDMS/DVB was the most efficient, followed by PA and CAR/PDMS/DVB. Thirty-eight compounds that comprise the aroma were identified among sesquiterpenes (56.4%) and monoterpenes (30.8%), such as *α*-fenchene, guaiol, globulol, *α*-muurolene, *γ*-himachalene, *α*-pinene, *γ*-elemene, and patchoulene.

## 1. Introduction

Cerrado is an ecosystem that concentrates one of the greatest biodiversities on the planet, occupying about 22% of the Brazilian territory and typified by a variety of plant species such as herbs, sub-shrubs, shrubs, trees, and vines, which together add up to more than 6000 identified species [1]. It is formed by different ecoregions due to its great latitudinal and longitudinal variation, comprising open pastures, savanna woods, forests, and perennial riparian forests [2]. Fruit trees stand out mainly due to their economic potential and the high nutrient content that they present in their compositions, in addition to the distinct flavor and aroma characteristics of their fruits [3].

Cerrado fruits such as araticum [4], grumixama [5,6], acerola [7], cagaita [8], murici [9,10], and pequi [11,12] have stood out commercially due to their unique nutritional properties, flavors, and aromas. The expansion of cultivation and advances in research on these fruits have fostered the development of new products for the food industry, providing income generation to communities of small producers and fruit collectors in the Cerrado [13,14,15].

*Eugenia klotzschiana* O. Berg belongs to the Myrtaceae family and has important nutritional value, including the content of polyphenolic compounds (566.3 mg Gallic Acid Equivalent 100 g^−1^) and flavonoids (550.0 mg Quercetin Equivalent/100 g), in addition to a considerable content of ascorbic acid (8.66 mg 100 g^−1^ fresh weight) and dietary fiber (6.45 g 100 g^−1^) [16,17]. The present study represents an advance in optimizing extraction conditions of volatile compounds from the fruits of *Eugenia klotzschiana* by the solid-phase microextraction (SPME) method. It is the first time that the influence of the fibers PA, DVB/CAR/PDMS, and PDMS/DVB, as well as temperature, time, and agitation employed in the extraction of VOCs present in these fruits were evaluated. In a previous study, one experimental condition used PDMS/DVB SPME fiber, allowing isolation of only 11 compounds [18].

Volatile compounds constitute the aroma of fruits, which is understood as the process of releasing low molecular weight compounds that impart odors and are captured by the human sensory system through olfaction [19]. It is noteworthy that the fruit aroma associated with the characteristics of color, texture, and size is fundamental in the perception and subsequent predilection of the consumer to a particular food. Furthermore, volatile compounds can be used to differentiate varieties of the same plant species [9], as important chemical signals in the cultivation and maintenance of the species in nature [20], and also allow the characterization of the fruit maturation stages and its quality as a whole [21].

Analysis of volatile compounds covers the process of extraction or recovery, desorption, separation, detection, and identification. Several extraction methods have been developed. Currently, headspace (HS) solid-phase microextraction (SPME) (HS-SPME) is used for the analysis of samples with complex matrices, such as food, due to the excellent results obtained and no employment of organic solvents or destruction of the analyzed sample. In addition to its repeatability and possibility to adjust the conditions and types of fibers used. Fibers available on the market vary in the type of coating and the thickness. Coatings represent the phase that establishes affinity for the analytes in the HS-SPME system, allowing their extraction, such as divinylbenzene/carboxen/polydimethylsiloxane (DVB/CAR/PDMS), polydimethylsiloxane/divinylbenzene (PDMS/DVB), and polyacrylate (PA). Desorption, separation, and detection steps can be performed using gas chromatography coupled with mass spectrometry (GC-MS) or high-performance liquid chromatography (HPLC) [9,22,23,24].

Therefore, the objective of this work was to study the optimal conditions for extraction, detection, and determination of volatiles from the pulp of *Eugenia klotzschiana* O. Berg fruits, as well as to define the best SPME fiber and discriminate the volatile chemical constituents employing HS-SPME and GC-MS.

## 2. Results and Discussion

**Figure 1:**
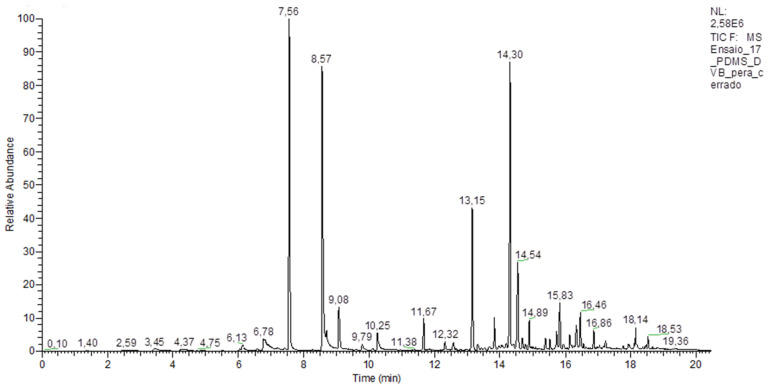
Peaks detected by GC-MS represent volatile compounds extracted by the PDMS/DVB fiber.

Major components of the aroma found in the pulp of *Eugenia klotzschiana* are sesquiterpenes (55.3%) and monoterpenes (31.6%). Esters, amines, and other compounds are present in smaller amounts (13.2%) (Figure 1). It is noteworthy that the volatile chemical composition is characteristic of each fruit due to the characteristics of the food matrix and the influence of external factors, such as environmental and cultivation conditions, to which the plant species was exposed [25].

In acerola, for example, volatiles are mainly made up of esters and alcohols [7], whereas for cambuí, sesquiterpenes correspond to 71% of the identified volatiles [9]. A study with cagaita (*Eugenia dysenterica*) showed monoterpenes representing 34.6% of the volatile profile and esters, 36.3% [26]. In contrast, grumixama (*Eugenia brasiliensis*) comprised 94.7% sesquiterpenes and 5.3% monoterpenes [5].

Thirty-eight volatile compounds were identified by employing three extraction fibers. Table A1 (Appendix A) shows the breakdown of volatile chemical constituents detected in the fruit pulp. The arranged columns represent identified compounds and the respective type(s) of fiber(s) that extracted them. The letter “X” marks the fiber that extracted the compound for identification purposes.

What is sensorially perceived as the aroma is the set of synergistic effects of volatile compounds present in fruits. The composition of the compounds becomes specific to each species [10,26]. However, it is possible to identify similar compounds in *E. klotzschiana* and cambuí, which have eight common volatiles in the aromatic profile: *α*-fenchene, guaiol, globulol, *α*-muurolene, *γ*-himachalene, *α*-pinene, *γ*-elemene, and patchoulene [27].

α-Pinene confers a characteristic odor of pine [28], also characteristic of the presence of camphene, which emits a woody and citrusy odor [29]. Myrtenol contributes to the odor of flowers and mint, while isogeraniol contributes to flowers and jasmine [30]. Linalyl acetate is responsible for the fruit and lavender notes, softening the fennel and lavanduol, the green lavender aroma [31]. *α*-Humulene is present in high amounts in hops (*Humulus lupulus*), which is used in beer production due to its flavoring potential. It provides a characteristic aroma to the beverage and other compounds and woody notes [29]. It is noteworthy that *E. klotzschiana* is consumed by the regional population in juices and is known as cervejinha do campo, “field beer”, due to the characteristic aroma of the fruit. Finally, guaiene and benzyl acetate contribute to the perception of wood and balsamic, apple, floral, and fruity notes, respectively [32].

**Figure 2:**
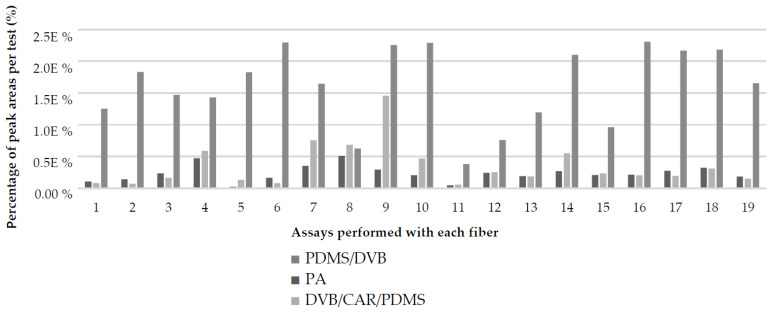
Percentage of peak areas of volatile chemical compounds extracted by SPME-HS using different types of coating.

Figure 2 shows the percentage of the area of volatiles extracted from the chromatograms using each SPME fiber. The largest volatile areas were detected in the chromatograms of the tests performed with PDMS/DVB fiber, followed by PA fiber and with the smallest area, DVB/CAR/PDMS fiber, corresponding to 73.4%, 15.9% and 10.7% of the entire identified area, respectively.

A possible interpretation of the fibers’ behavior due to the volatiles present in the pulp of *E. klotzschiana* is the affinity of the type of coating of each fiber for specific analytes since the aromatic profile of *E. klotzschiana* is mainly composed of sesquiterpenes (56.41%), which are compounds characterized by medium polarity. The PDMS/DVB coating is semipolar, making it chemically more susceptible to adsorb substances with similar polarity [10]. While esters, which have higher polarity, represent less than 13.60% of the volatiles, corroborating the values of the area extracted by the fiber with the polyacrylate coating.

The DVB/CAR/PDMS fiber had the smallest area extracted from volatiles. However, five compounds were only isolated in assays carried out with this coating: cadina-3,9-diene, (±)-*α*-pinene, *γ*-himachalene, (±)-camphene, and sabinene hydrate trans acetate.

### 2.1. Evaluation of the Effect of Independent Variables on the Extraction of VOCs

#### 2.1.1. Extraction Temperature

Temperature is one of the main factors influencing the yield of volatile compounds’ extraction, especially in plant matrices [7,9,26]. This condition is due to its ability to maximize or reduce the extracted volatiles due to the potential degradation of analytes that exacerbated temperature values can trigger [33]. For the levels used in this study, the temperature significantly affected the experiment performed with DVB/CAR/PDMS and PDMS/DVB fibers.

**Figure 3:**
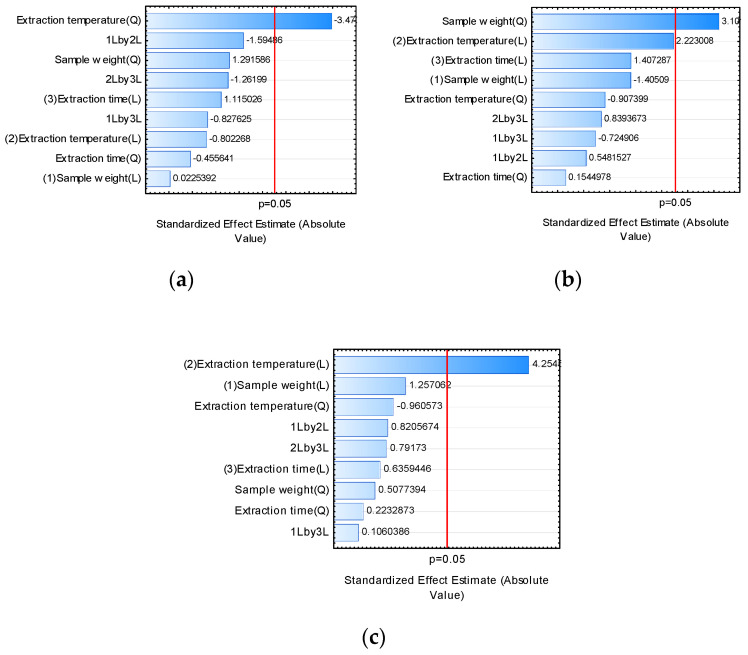
Effects of time and temperature of extraction and quantity of pulp variables on the extraction of volatiles using different fiber coatings for HS-SPME: (**a**) PDMS/DVB, (**b**) PA, and (**c**) CAR/PDMS/DVB.

As for DVB/CAR/PDMS fibers, the temperature was the only variable that exerted a significant effect, maximizing the area of extracted volatiles (Figure 3). It was observed that the largest extraction areas were obtained at temperatures from 80 °C. Related to the region with the most significant area, the use of larger amounts of pulp is noted, indicating that, in order to obtain significant values for the volatile area, it is necessary to use high temperatures and a larger quantity of pulp. A possible explanation is the lower affinity of the compounds with the fiber coating, making it demand high values of the system variables to obtain a better response [22].

Observing the graph of the response surface of the PDMS/DVB fiber (Figure 3), which depicts the behavior of temperature in the extraction of volatiles, it appears that temperature had a positive effect on the area of extracted volatiles. Thus, the response was maximized in the presence of heat, especially from 50 to 107 °C, where the region of the response surface was identified with a larger area. However, temperatures higher than 107 °C reduced the volatile area, indicating that although it is effective and important for the HS-SPME system in the analyzed sample, exacerbating values can negatively affect volatile extraction. A possible explanation for this is the occurrence of degradation of volatiles due to the use of high temperatures [33], which in the studied system was temperatures higher than 107 °C. From 50 °C, a positive effect on the extraction yield is already observed, indicating that applying high temperatures is unnecessary.

In assays carried out with PA fibers, the temperature conditions had no significant effect on the extraction of volatiles. The lowest temperature values were enough to release analytes from the plant matrix and their adsorption by compatibility with the polyacrylate coating from the headspace. This reinforces that it is not necessary to use high-temperature values to maximize the yield of volatile extraction from *E. klotzschiana* in the studied systems.

#### 2.1.2. Extraction Time

Extraction time is an essential parameter for the effective use of the HS-SPME technique. It must be sufficient to release the analytes from the analyzed matrix until they settle in equilibrium in the headspace vial and are adsorbed or absorbed by the fiber coating [23,33,34]. In the experiments carried out, ranging from 10 to 30 min, the time did not significantly affect the volatile extraction yield for the studied fibers. 

**Figure 4:**
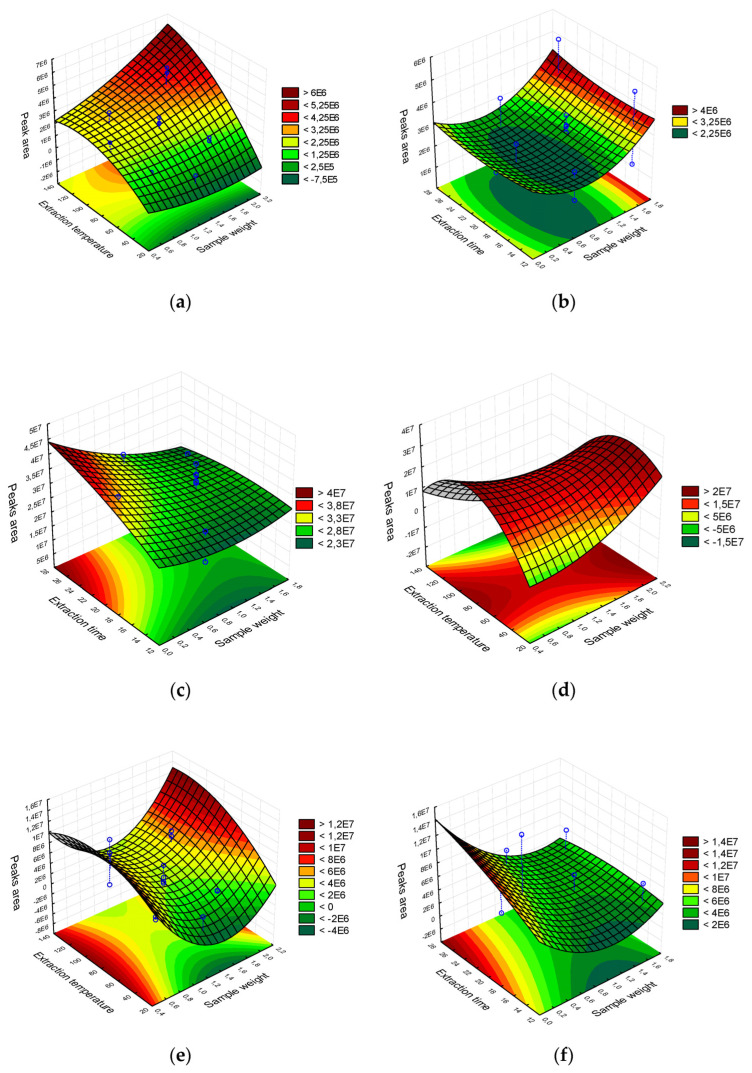
Three-dimensional response surface (RSM) graphs of the variables time and temperature of extraction and amount of pulp in the extraction of volatiles using different fiber coatings for HS-SPME: (**a**) DVB/CAR/PDMS extraction temperature vs. sample weight, (**b**) DVB/CAR/PDMS extraction time vs. sample weight, (**c**) PDMS/DVB extraction time vs. sample weight, (**d**) PDMS/DVB extraction temperature vs. sample weight, (**e**) PA extraction temperature vs. sample weight, and (**f**) PA extraction time vs. sample weight.

Observing the DVB/CAR/PDMS fiber response surface graph (Figure 4), it is possible to confirm no influence of weather conditions on the extracted volatile area. The most significant areas were obtained from 24 min of fiber exposure. It was observed that there was no increase in the efficiency of extracting volatile compounds from the sample, even in tests with more extended time values at a constant sample quantity.

On the other hand, the volatile areas extracted by PDMS/DVB were greater when using time values greater than 20 min, even when associated with a smaller amount of sample, and intrinsically, a lower concentration of VOCs. In regions with sample amounts greater than 2.0 g, it is also possible to obtain higher extraction yields. However, they were not superior to those obtained with 0.5 g of *E. klotzschiana* pulp at a time longer than 20 min. The optimal extraction time calculated by the model was 19.71 min, confirming what was demonstrated by the response surface.

As for the PA fiber, it was observed that the highest extraction yield values were detected in a time greater than 20 min. However, similar values were not observed when larger amounts of sample were used, as occurred in the PDMS/DVB fiber. A possible explanation is that the fiber sorbent reached its maximum adsorption capacity of the analytes to which it was exposed [35].

#### 2.1.3. Sample Weight

Sample weight in the experiments ranged from 0.5 to 2.0 g of *E. klotzschiana* pulp. The volume contained in the headspace flask must be sufficient for the isolation of volatiles and their adsorption and/or absorption by the sorbent to occur [9].

It was found that sample weight did not influence volatile extraction yield when using DVB/CAR/PDMS and PDMS/DVB fibers. However, it is noteworthy that, due to the larger sample volume, it was possible to detect an increase in yield in PDMS/DVB assays that used more than 2.0 g of pulp with extraction times shorter than 16 min. This result portrays that a greater quantity of analytes were eluted into the vapor phase (headspace) due to the more significant amount of pulp, and the fiber could adsorb this greater fraction, maximizing the extracted area.

When analyzing results with DVB/CAR/PDMS fibers, the most considerable areas were obtained only from 1.4 g of pulp, regardless of the temperature or time employed.

As for PA fibers, the variation in pulp amount positively affected the yield of extraction of volatiles, increasing the area extracted when the amount of pulp was more significant than 2.0 g. However, it is essential to emphasize that 0.5 g was enough to obtain the same volatile areas. What happens when using 2.0 g of pulp or more is the greater accumulation of analytes in the headspace due to the more significant amount of substrate, so that the content was able to be absorbed by the polyacrylate sorbent, for the volatiles present in the pulp of *E. klotzschiana* at the levels and parameters studied [34].

## 3. Materials and Methods

### 3.1. Sample Acquisition

Fruit samples *of Eugenia klotzschiana* O. Berg were obtained through a donation from trees located in the region of Turmalina, State of Minas Gerais, Brazil (Lat-17.287410, Long-42.718210). Fully ripened fruits (greenish-yellow skin color) were collected directly from trees from January to February 2019, amounting to about 3 kg.

After collection, the fruits were washed under running water to remove impurities and sanitized with a chlorinated solution (200 ppm) for 15 min, followed by rinsing in running water. The fruits were then fully packed in polyethylene bags and stored at a temperature of −18 °C until analysis. Samples were transported in a secondary package containing ice to the Mass Spectrometry Laboratory of the Department of Chemistry at the Federal University of Minas Gerais (UFMG), Belo Horizonte, Minas Gerais, Brazil.

Analyses were performed using the fruit pulp, obtained by manual pulp removal, followed by homogenization with a mixer (Mixer Mondial Versatile Black M-08).

### 3.2. Experimental Design

The central composite design was used with five repetitions at the central point and six axial points, with three independent variables: extraction time (minutes), extraction temperature (Celsius), and sample weight (grams), with two levels each, namely: for extraction time a minimum level of 10 min, a maximum of 30 min, and a center point of 20 min, for extraction temperature a minimum of 30 °C, a maximum of 120 °C, and a center point of 75 °C, and for sample weight a minimum of 0.5 g, a maximum of 2.0 g, and a center point of 1.25 g.

Three types of fibers were used, DVB/CAR/PDMS (50/30 μm), PDMS/DVB (65 μm), and PA (85 μm) (Sigma-Aldrich, St. Louis, MO, USA) [24]; thus, three experiments were performed with nineteen repetitions each.

Peaks included were those with a relative abundance greater than 2% of the total area of the chromatogram. The total relative area (%) of the peaks: the sum of the percentage abundance of the area of valid peaks obtained in the chromatograms, was determined as the dependent variable using Microsoft Office Excel^®^ 2010. Results were analyzed according to the area extracted by each fiber and the behavior of the independent variables through the response surface methodology using Statisticv.10 (Stat-Soft Inc., Tulsa, OK, USA).

### 3.3. Headspace Solid-Phase Microextraction (HS-SPME) 

*Eugenia klotzschiana* O. Berg pulp was transferred to identified glass vials. Pulp amount was weighed according to the experimental planning, and the flasks were sealed with an aluminum seal and septum rubber [7,10].

Subsequently, the vials containing the pulp were subjected to time, temperature, fiber type, and extraction conditions. For this purpose, a system was structured containing a heating plate, an aluminum block to contain the vials with the sample, and an iron support with a clamp to ensure the proper positioning of the SPME device containing the fiber when exposed. After extraction, the fiber was retracted and taken to the chromatograph (GC-MS) for desorption of the compound [9,23,34,36].

### 3.4. Gas Chromatography Coupled with Mass Spectrometry Analysis and Identification of Volatile Compounds

Volatile compounds were analyzed using a gas chromatograph (Trace CG Ultra) coupled to the mass spectrometer (Polaris Q) with an ion-trap analyzer (Thermo Scientific, San Jose, CA, USA). Compounds were separated using a capillary column HP−5 MS (5% phenyl and 95% methylpolysiloxane) of 30 m long, 0.25 mm internal diameter, 0.25 μm film thickness, and helium gas with a constant flow rate of 1 mL min^−1^ (Agilent Techonolgies Inc., Waldbronn, Germany). The injector (splitless mode) was maintained for 5 min at a temperature of 250 °C, the ion source at 200 °C, and the interface at 270 °C. The oven was programmed at 40 °C for 1 min, followed by an increase in temperature at a rate of 12 °C min^−1^ until it reached 120 °C, maintaining it for 2 min. Then, at 15 °C min^−1^ to 150 °C and finally, at 20 °C min^−1^ to 245 °C, maintaining for 2 min.

The identification of volatile compounds was carried out using the National Institute of Standards and Technology Research Library (NIST). This identification was also based on articles that determined volatile compounds in *Eugenia klotzschiana* O. Berg. The total peak area was obtained in Xcalibur1.4 from Thermo Electron Corporation (Thermo Electron, San Jose, CA, USA) and analyzed in Microsoft Office Excel 2010^®^.

## 4. Conclusions

The profile of volatile compounds from the pulp of *Eugenia klotzschiana* O. Berg is varied and complex, containing 38 volatiles constituted by 55.3% of sesquiterpenes and 31.6% of monoterpenes. The PDMS/DVB fiber allowed the identification of 23 volatiles, the PA fiber 17, and the DVB/CAR/PDMS fiber only 8. Variables of time, temperature, and weight of the sample behaved differently for the three fibers studied. However, the HS-SPME method proved to be effective in extracting volatile compounds present in the pulp of *Eugenia klotzschiana* O. Berg, with the best performance occurring when using the PDMS/DVB fiber, under conditions of 56 °C, 2.6 g of pulp, and 20 min of extraction. Consequently, this study presented new information about a fruit species of great importance in the Cerrado.

## Data Availability

All data are contained within the article.

## Sample Availability

Samples of *Eugenia klotzschiana* O. Berg are available from the authors.

## Appendix A

**Table A1:** Volatile organic compounds identified by three different types of SPME fibers and GC-MS in the pulp of *Eugenia klotzschiana* O. Berg.

N.	Name	Formula	CAS	Reference	PA	PDMS/ DVB	CAR/PDMS/DVB
**Monoterpenes**							
01	(±)-α-pinene	C_10_H_16_	80-56-8	[10]			X
02	p-mentha-1,8-dien-7-ol	C_10_H_14_O	536-59-4	[37]		X	
03	(2S, 5S)-2-methyl-5-propan-2-ylbicyclo [3.1.0] hexan-2-ol	C_10_H_18_O	------------			X	
04	myrtenol	C_10_H_16_O	515-00-4	[30]		X	
05	p-mentha-3,8-diene	C_10_H_16_	586-67-4	[38]		X	
06	(±)-camphene	C_10_H_16_	79-92-5	[29]			X
07	α-fenchene	C_10_H_16_	471-84-1	[10]	X	X	
08	linalyl acetate	C_12_H_20_O_2_	115-95-7	[31]			X
09	ocimene	C_10_H_16_	29714-87-2	[31]	X	X	
10	(−)-lavandulol	C_10_H_18_O	498-16-8	[31]	X		
11	sabinene hydrate trans acetate	C_12_H_20_O_2_	------------	------------			X
12	isogeraniol	C_10_H_18_O	5944-20-7	[30]	X		
**Sesquiterpenes**							
13	δ-elemene	C_15_H_24_	20307-84-0	[10]	X	X	
14	isoledene	C_15_H_24_	95910-36-4	[21]		X	
15	β-guaiene	C_15_H_24_	88-84-6	[39]		X	
16	α-humulene	C_15_H_24_	------------	[29]	X	X	
17	(+)-calarene	C_15_H_24_	17334-55-3	[40]		X	X
18	cadinene	C_15_H_24_	29350-73-0	[21]		X	
19	viridiflorene	C_15_H_24_	21747-46-6	[10]		X	
20	α-guaiene	C_15_H_24_	3691-12-1	[10]	X	X	
21	γ-himachalene	C_15_H_24_	53111-25-4	[10]			X
22	(−)-γ-muurolene	C_15_H_24_	24268-39-1	[10]	X	X	
23	acoradiene	C_15_H_24_	24048-44-0	[41]	X	X	
24	(+)-cyclosativene	C_15_H_24_	22469-52-9	[21]	X	X	
25	(−)-caryophyllene	C_15_H_24_	87-44-5	[21]		X	
26	cadina-3,9-diene	C_15_H_24_	523-47-7	[42]			X
27	globulol	C_15_H_26_O	489-41-8	[10]	X	X	
28	patchoulene	C_15_H_24_	-----------	[10]	X		
29	α-caryophyllene alcohol	C_15_H_2_6O	4586-22-5	[43]	X		
30	(−)-guaiol	C_15_H_26_O	489-86-1	[10]	X		
31	cedryl acetate	C_17_H_28_O_2_	77-54-3	[44]	X		
32	2-naphthalenol,2,3,4,4alpha,5,6, 7-octahydro-1,4alpha-dimethyl-7-(2-hydroxi-1-methylethyl)	C_15_H_26_O_2_	-----------	-----------	X		
33	spiro [4.5]decan-7-one, 1,8-dimethyl-8,9-epoxi-4-isopropyl	C_15_H_24_O_2_	61050-91-7	[20]		X	
**Other**							
34	N-(2-hydroxipropyl) ethylenediamine	C_15_H_14_N_2_O	123-84-2	-----------		X	
35	ethyl hexanoate	C_8_H_16_O_2_	123-66-0	[45]		X	
36	benzyl alcohol	C_7_H_8_O	100-51-6	[45]	X		
37	benzyl acetate	C_9_H_10_O_2_	140-11-4	[46]	X	X	X
38	(E)-3,7-dimetlnon-6-enal	C_11_H_20_O	-----------	-----------		X	
	Total compounds identified by each fiber				18	23	08

CAS (American Chemical Society), divinylbenzene/carboxen/polydimethylsiloxane (DVB/CAR/PDMS), divinylbenzene/polydimethylsiloxane (PDMS/DVB), polyacrylate (PA).
